# Flavescence Dorée and Grapevine Susceptibility: From Host–Pathogen Interaction to Cultivar Categorization

**DOI:** 10.3390/pathogens14090939

**Published:** 2025-09-16

**Authors:** Alessandro Bene, Marzia Vergine, Athos Pedrelli, Luigi De Bellis, Andrea Luvisi

**Affiliations:** 1 Department of Biological and Environmental Sciences and Technologies, University of Salento, 73100 Lecce, Italy; alessandro.bene@unisalento.it (A.B.); athos.pedrelli@unisalento.it (A.P.); luigi.debellis@unisalento.it (L.D.B.); andrea.luvisi@unisalento.it (A.L.); 2 National Biodiversity Future Center, 90133 Palermo, Italy

**Keywords:** *Vitis vinifera*, FD phytoplasma, Grapevine Yellows disease, *Scaphoideus titanus*, cultivar classification

## Abstract

Flavescence dorée (FD) is a major grapevine disease in Europe with significant economic consequences. The objective of this review is to provide as much information as possible on the documented susceptibility or tolerance of different cultivars, both international and local within individual countries. Additionally, spontaneous recovery has been observed as a viable option for replanting injured vines, but its efficacy varies by cultivars. In this regard, a broad categorization was developed for several cultivars, particularly those examined in Europe, describing their higher or lower susceptibility and aptitude to recover. Future research, however, should not only address the geographic spread of FD, but also investigate how pathogen–host interactions may differ across cultivars. Such insights could be crucial for assessing the risk of FD introduction in new regions and understanding cultivar-specific susceptibility and epidemic dynamics, because present studies remain mostly concentrated in regions suffering significant FD pressure, resulting in a focus on a small number of cultivars that are often specific to a certain geographical area. Furthermore, the implementation of innovative strategies has the potential to give a comprehensive and long-term approach to managing and containing FD.

## 1. Flavescence Dorée and Its Distribution

The grapevine (*Vitis vinifera* L.) is a globally significant crop, covering over 7.2 million hectares [1]. In this regard, grapevine diseases receive particular attention due to their significant economic impact [2]. Among the various microorganisms that can affect the health and yield of the grapevine, phytoplasmas stand out as a serious and damaging disease [3]. Phytoplasmas are prokaryotic organisms without cell walls, classified within the Mollicutes class [4], and with a range in size from 200 to 800 nm [5]. These microorganisms are phylogenetically related to the Gram-positive bacteria [6]. Phytoplasma cells appear as vesicular, rounded structures that move within phloem sieve tubes, passing through sieve plate pores. However, their movement is sluggish, and their spread throughout the plant is unpredictable, infecting all plant organs, including roots, canes, shoots, buds, flowers, and berries, but not seeds [7]. They can exhibit polymorphism and can only survive and reproduce in isotonic environments, such as plant phloem or insect haemolymph within phloem-sucking leafhoppers, planthoppers and psyllids [8]. Consequently, they are entirely dependent on their hosts, but can replicate within insect vectors and, in some cases, even infect their eggs [9].

Among the main damages they cause to the plant is the alteration of the functionality of the phloem sieve tubes [10]. The phytoplasma chromosome is very small (680–1600 kb), and efforts to grow phytoplasmas in cell-free media have been unsuccessful [9]. However, with the advancement of molecular techniques, it was possible to identify a wide range of phytoplasmas causing diseases in numerous plant species [11]. As a result, phytoplasmas are categorized into over 30 groups based on their 16S rRNA sequences within the taxon *Candidatus* Phytoplasma [12].

Various Grapevine Yellows (GY) diseases linked to phytoplasmas are recognized in many grape-producing countries, where they contribute to crop loss and a decline in quality [13]. The most important GY diseases in the main viticultural areas of Europe are “Flavescence dorée” (FD) and “Bois noir” (BN) [14], previously mentioned as a provisional taxon known as “*Candidatus* Phytoplasma vitis” [15], and “*Candidatus* Phytoplasma solani” [16], respectively. They produce similar symptoms, such as abnormal lignification of canes, shortened internodes, flower abortion, leaf discoloration and curling, as well as yellowing or reddening, depending on the variety [17]. The diseases mainly differ in their epidemiological patterns, such as severity and progress of the attack in vineyards, vectors and source of inoculum [18]. GY diseases caused by different phytoplasmas can occur in the same region or even within the same vineyard, as seen with the GY diseases in Central-Southern Europe [19]. Therefore, identifying the phytoplasmas in a symptomatic grapevine cannot be conducted through visual inspection alone and requires laboratory diagnostic techniques [3]. Several other diseases similar to FD and BN have been observed and researched in numerous countries [20,21]. GY diseases are caused by different phytoplasma species and groups, and involve distinct insect vectors that either specifically or occasionally feed on the vines [22].

In detail, FD phytoplasma (FDp) belong to two taxonomic subgroups namely 16SrV-C and 16SrV-D, and so to three phylogenetic strain clusters, according to multilocus sequence analysis of map, vmpA, uvrB-degV and secY loci [23,24,25]. FD phytoplasma can be transmitted from vine to vine by the ampelophagous, monovoltine leafhopper *Scaphoideus titanus* [26]. This leafhopper is highly suited to viticultural regions where the summer season is long enough for the adults to lay their eggs [18]. While *S. titanus* is the primary vector responsible for epidemic spread in vineyards, recent studies have identified *Dictyophara europaea*, a polyphagous planthopper prevalent in natural habitats, as an important alternative vector capable of maintaining the pathogen outside cultivated areas [27]. FD is therefore an epidemic, economically important, quarantine disease of grapevine in Europe [17]. It often occurs as an epidemic because it affects a significant number of vineyards within a region, with up to 95% of the grapevines in an individual vineyard showing signs of infection [19].

The first outbreak of FD was reported in 1955, in Armagnac, France, where it was assumed to be a form of root asphyxia [28,29], and the disease has since dispersed to other European winegrowing regions such as Italy, Portugal, Spain, Serbia, Slovenia, Switzerland, Hungary, Croatia and Austria [30]. Lastly, in recent decades, there has been a significant expansion of vine-growing areas in countries such as China, India, Japan, Korea, Thailand, and Indonesia. This growth has heightened the risks associated with the introduction of grapevine phytoplasma diseases, which could pose a threat to vineyard ecosystems and the grape industry in these Asian nations [31].

All species within the genus *Vitis* (*V. riparia*, *V. labrusca*, *V. longii*, *V. simpsonii*, *V. doaniana*, *V. champinii*, *V. amurensis*, *V. rubra*, *V. rupestris*, *V. pentagona*, *V. sylvestris*, *V. vinifera*), as well as their hybrids used as rootstocks, are susceptible to FD [32]. The aim of this review is to provide an up-to-date overview of the susceptibility and tolerance of different Vitis vinifera cultivars to Flavescence dorée, investigating the host–pathogen relationship and the plant’s ability to recover, based on the literature, and to establish a practical and useful classification of these cultivars.

## 2. Host–Pathogen Interaction and Resistance Factors

### 2.1. GY Symptoms

**Figure 1:**
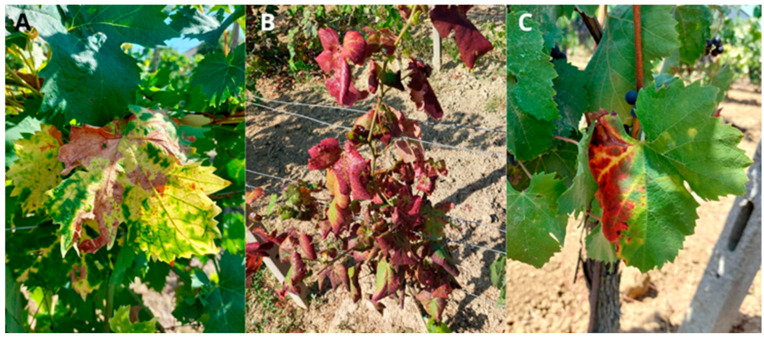
Foliar symptoms of Flavescence dorée (FD) in different grapevine cultivars. (**A**) Reddening of leaves in a red Yellowing and leaf curling in a white cultivar. (**B**) Intense reddening and pronounced downward curling of the leaf blades in a red cultivar, indicative of advanced phloem dysfunction. (**C**) Interveinal reddening and marginal necrosis in another red cultivar, reflecting vascular blockage and tissue degeneration.

As previously mentioned, GY are diseases that share similar symptoms, which makes it difficult to identify the specific phytoplasma causing the infection just by observation [33]. FDp leads to considerable damage to the plant, presenting symptoms that stem from damage to the phloem and vascular cambium in the shoots and trunk [34]. This occurs because the phytoplasma interferes with local hormonal balance and carbohydrate transport, particularly in the phloem, leading to altered signaling that promotes vegetative growth over reproductive development [35]. FD symptoms are typically observed on leaves, clusters, and canes, usually appearing one year after the infection [36] (Figure 1). For infected vines, symptoms generally emerge around mid-summer (mid-July in the boreal hemisphere; mid-January in the austral hemisphere), and worsen progressively throughout the growing season, becoming easily noticeable [37]. Key symptoms include abnormal lignification of canes, the appearance of blackish pustules on the shoots, shortened internodes, flower abortion, leaf discoloration, curling, and varying yellowing or reddening, also affecting the veins, depending on the variety [38]. The leaves of affected shoots are stiff, curled downward, and range in color from metallic yellow to yellow-green or green, with chlorotic patches that eventually become necrotic. Leaves on the basal and middle parts of shoots sometimes fall off mid-season [39]. The veins are generally stiff, fragile, and curl downward [40]. Diseased shoots are limp and rubbery, with short internodes and zigzag growth, often staying green or grayish-green, while healthy shoots develop brown periderm [39]. When the first symptoms appear, the disease causes significant deterioration throughout the plant, including severe damage to the inflorescences. If the symptoms appear after flowering, flower abortion can occur [35]. Clusters on affected shoots may abort near bloom or the rachis and berries shrivel before harvest [39]. Grapes that are harvested at the end of the season are underdeveloped and unsuitable for winemaking, as their juice is sour and lacks sugar. As a result, much of the grape yield is lost [35]. Other distinct symptoms include abnormal growth of shoots and roots, etiolation, and necrosis of the vascular cambium and phloem [40]. Over time, there is a general decline in the health of the plants. However, plants with partial infection may still produce a good yield and can survive for many years [36].

### 2.2. Colonization and Plant Resistance Mechanisms

Phytoplasmas have a distinctive and intricate life cycle, involving colonization of multiple environments, including the plant phloem and various organs of phloem-feeding homoptera insects [41]. In plants, these microorganisms predominantly reside in phloem components, such as mature sieve tubes and developing phloem cells. Due to their lack of a cell wall, phytoplasmas must regulate their internal osmotic pressure to match that of the sieve elements [42]. In insects, they need to pass through the gut cells, replicate in different tissues, and reach the saliva, where they can be transferred into plants [43]. Phytoplasmas, which are localized in the phloem sieve tubes of the phloem, invade the phloem- conducting organs, such as veins, leaf petioles, and shoots. Their colonization pattern, observed in different cultivars such as Chardonnay and Refoscod’Istria, is considered similar [19]. This invasion disrupts the descending vascular system, leading to the blockage of lymphatic tubes and medullary tissues [19], which ultimately interrupts the transport of photosynthates [4]. Since photoassimilates cannot be stored in other plant parts outside the leaves, starch accumulation occurs, which is believed to contribute to the blockage of phloem sieve tubes [19]. Consequently, starch accumulates in the leaves, hindering the transport of processed substances and progressively affecting the nutrition of the grapevine, shoots, and stems, as found on the hybrid “Baco 22 A” [35]. After entering the phloem sieve tube elements, phytoplasmas spread throughout the plant by passing through the phloem sieve plate pores. Occasionally, adjacent phloem parenchyma and companion cells are also invaded [44], though their entry mechanism remains unclear. It seems unlikely that phytoplasmas pass through pore-plasmodesmata units, as these have pore diameters of only 3–4 nm [45].

Phytoplasmas possess two secretion systems: the YidC system, responsible for integrating membrane proteins, and the Sec system, which integrates and secretes proteins into the host cell cytoplasm [46]. One of the earliest physiological effects of infection is believed to be the disruption of auxin gradients and other hormonal signaling pathways within the phloem. This hormonal imbalance leads to altered carbon allocation and developmental anomalies [34]. The data indicate that phytoplasma infections significantly disrupt key primary metabolic processes, such as glycolysis, the tricarboxylic acid (TCA) cycle, and amino acid metabolism, as observed in recovered Barbera plants. These pathways are essential for cell survival and provide intermediate and end products that participate in various metabolic pathways [34]. Alterations in photosynthate movement, alongside other physiological disturbances like reduced photosynthesis, pigment levels, stomatal conductance, transpiration, root respiration, and hormonal imbalances, likely contribute to the symptoms observed in infected plants [47]. The decline in photosynthetic activity and subsequent changes in carbohydrate metabolism impact the synthesis of carbohydrates, chlorophyll (resulting in leaf yellowing), and carotenoids [48]. Similarly, microarray analysis of BN phytoplasma-infected Nebbiolo leaves revealed downregulation of genes encoding photosystem I and II proteins and a reduction in Rubisco activase expression [2]. In line with the findings of Margaria and Palmano [2], a photosystem II component (Mn-stabilizing protein) was downregulated, while cysteine synthase, a key player in primary metabolism, was upregulated. Cysteine is involved in glutathione (GSH) biosynthesis, a molecule that participates in multiple pathways including redox signaling and modulation of auxin sensitivity, thereby contributing to the plant’s defense and development under stress conditions [49].

Many of the proteins altered during FD infection belong to the “cell rescue, defense, and virulence” category, such as “pathogenesis-related proteins” [50]. This is further supported by Margaria et al. [34], who found increased expression of pathogenesis-related proteins in infected tissues of Barbera grapevine. Additionally, heat shock proteins, essential for developmental processes and stress responses, were identified as stress-responsive proteins [51]. In agreement with their findings, Rubisco and other proteins involved in photosynthesis and carbon metabolism were phosphorylated, suggesting that phosphorylation might signal protein degradation, impair the photosynthetic machinery, and disrupt electron transfer, contributing to the generation of reactive oxygen species (ROS) in infected leaves [52]. Consequently, Oliveira et al. [53] observed in cv. Loureiro a reduction in leaf area by up to 60%. The phytoplasma genome also contains the *sodA* gene, which encodes the Mn-SOD protein capable of neutralizing ROS, a key component of the plant’s defense response [54]. Also, phytoplasmas may exacerbate symptoms by secreting effector proteins and modifying plant gene expression. For instance, the TENGU gene, which suppresses signaling or biosynthesis of auxin indole-3-acetic acid (IAA), has been linked to symptoms such as witches’ broom and dwarfism [55]. In response, there are two primary mechanisms by which plants defend themselves against these pathogens: resistance, which refers to the plant’s ability to restrict pathogen growth, and tolerance, which is the plant’s ability to mitigate the impact of infection on its overall fitness, regardless of how much the pathogen multiplies [56]. But the essential step to trigger the defense is generally the recognition of the pathogen: initially, they detect general elicitors (PAMPs: Pathogen-Associated Molecular Patterns), which are structural components of the pathogen’s cell wall or nucleic acids [57], or endogenous elicitors (DAMPs: Damage-Associated Molecular Patterns), which are signaling molecules produced by the infected plant [58]. Subsequently, the plant identifies specific pathogen proteins, known as effectors [59]. These effectors are recognized by R-receptors, triggering a more elaborate and intensified defense response, referred to as Effector-Triggered Immunity (ETI). This leads to the establishment of a hypersensitive reaction, reducing the pathogen’s virulence and limiting its threat to the plant [60].

Grapevine varieties exhibit varying degrees of susceptibility to FD even when grown under the same environmental conditions and exposed to the same disease pressure [36]. The study of these aspects is also made difficult by the restrictions established by legislation (e.g., the European one, (UE) [61] which require the uprooting of infected plants. For this reason, many studies are related to BN rather than FD, despite the awareness that there cannot be a perfect analogy between the two responsible microorganisms [62]. Furthermore, assessing cultivar susceptibility in field conditions is challenging due to uncontrolled environmental factors that influence the presence and abundance of *Scaphoideus titanus*, infection pressure, and the pathways through which the pathogens enter the vineyard (wind direction, altitude, vineyard slope) [63]. Therefore, studying resistance factors is also tricky and sometimes specific phenomena such as recovery are investigated to understand some factors predisposing to resistance. Recovery refers to the phenomenon where plants show spontaneous and stable remission of symptoms in the following growing season, accompanied by the disappearance of the pathogen from the canopy and the restoration of grape productivity, as observed in cvs. Barbera and Albarola [64,65]. This recovery is thought to be linked to the down-regulation of ascorbate peroxidase, which likely leads to a long-term accumulation of hydrogen peroxide (H_2_O_2_) in plant tissues. This accumulation may help counteract the pathogen’s virulence and facilitate recovery [34]. However, the reasons behind the differing susceptibility of cultivars remain difficult to identify and require further study. One hypothesis suggests that the plant’s proteomic response may play a role [2]. For instance, Nebbiolo plants exhibit an abundant presence of proteins associated with “cell rescue, defense, and virulence” during infection, while Barbera shows a lower presence of these proteins [66].

## 3. Susceptibility of Main Grapevine Cultivars

The expression of symptoms, phytoplasma concentration, and the incidence of infected plants differ across grapevine cultivars [66]. However, it is difficult to have consolidated data following comparative evaluations between different cultivars due to the before mentioned legislative restrictions that affect many countries involved in FD epidemics and that require, above all, experiments in conditions of natural infection. Therefore, only evaluations (comparisons between FD-positive and FD-negative plants of a single cultivar) or comparisons between two cultivars are frequently reported [67]. Although the presence of FD in an area could affect more vineyards, generally the outbreak is quickly identified, not very extensive, and rarely affects numerous vineyards of different varieties [19]. However, indirect comparisons can be made by considering rather similar experiments, thus defining cultivars considered more or less susceptible.

Varieties such as Chardonnay and Pinot Gris exhibit severe symptoms, while others like Tocai Friulano and Moscato Bianco generally show symptoms in only a few plants or branches, displaying low susceptibility to FD and a strong ability to recover [36]. The primary plant hormones involved in modulating inducible defenses are salicylic acid (SA) and jasmonic acid (JA), involved in a reciprocal antagonism [68]. Studies investigating plant-phytoplasma interactions have consistently reported an up-regulation of SA-signaling, during infections caused by FD [33], while JA-mediated defense responses are suppressed [69]. This SA-JA crosstalk may explain the differences in susceptibility to FD observed between Chardonnay and Tocai Friulano [36]. Similarly, Albarola is less prone to FD and recovers better than Vermentino, where plants often die, as noted by Boselli [64]. Also, a study in vineyards revealed that Barbera was more susceptible to FD compared to Nebbiolo, showing about one order of magnitude higher phytoplasma titres [66].

Another interesting example from Piedmont is a hierarchical classification of local grapevine cultivars proposed by Ripamonti et al. [63], which ranks them from the least to the most susceptible to FD. In this classification, Moscato, Brachetto, Merlot and Freisa are grouped together as having the lowest susceptibility, followed by an intermediate group with Nebbiolo, Arneis, Timorasso and Erbaluce, and then the most susceptible such as Dolcetto, Barbera NC, Cortese, Barbera 84 and Ruché. Wine production in Piedmont traditionally involves various grapevine cultivars [70]. The potential for highly susceptible grapevine varieties to enhance vector transmission efficiency, thereby influencing disease epidemiology, is becoming an important issue to address for managing FD in this region [71]. Generally, varieties grown in FD-affected regions across France, Italy, and Spain vary in their susceptibility. Some of these cultivars have been classified based on the severity of symptoms and their ability to recover from FD (Table 1). Sangiovese, Garganega, Perera, Plovdina, Frankovka, Istrian Malvasia and Loureiro are highly sensitive and typically do not recover after infection. Grenache Noir, Barbera, Cabernet Franc, Cabernet Sauvignon, Sauvignon Blanc, Chardonnay, Riesling, Trebbiano, Pinot Gris, Zupskibojadiser, Smederevka, Black Burgundy, Italian Riesling and Glera are also sensitive, but may recover if protected from further inoculations [19,30,72]. Varieties such as Merlot, Pinot Noir, Erbamat, Croatina and Nebbiolo are more tolerant, although heavily infected vines can still be found. Symptoms are rare in Syrah, Teran and Magdeleine [73,74,75,76,77].

In terms of rootstocks, a study by Eveillard et al. [78] examined several rootstocks, including 110 Richter, 3309 Couderc, 41 B, Kober 5BB, Nemadex, Riparia Gloire de Montpellier, and SO_4_. Except for Kober 5BB and Nemadex, which were classified as poorly susceptible, all other rootstocks were grouped in the “intermediate susceptibility” category.

However, excluding cases such as Chardonnay and Riesling, which are generally the most susceptible to FD, it remains difficult to have a clear hierarchy among cultivars worldwide, as the more or less intense response of the plant to the disease may depend greatly on the surrounding environment, thus finding different susceptibility scales and cultivars depending on the areas considered [19]. Therefore, we can only obtain a more specific view by restricting the field to a single country or geographical area of the world, as reported in some of the studies cited above. Furthermore, it is difficult to correlate the susceptibility of cultivars to a single genotype. In the literature, it is possible to find information on individual genotypes depending on different areas or countries, but much more difficult depending on the specific cultivar. In the cultivars of the previously mentioned studies, only the subgroups 16SrV-C and -D, within the 16SrV taxonomic group, were identified.

As previously mentioned, recovery is a phenomenon characterized by the remission of disease symptoms after a prior infection, also observed in plants infected with phytoplasmas [36]. Therefore, recovery from FD, when integrated with conventional control methods, could serve as a viable strategy for managing the disease, particularly in long-established vineyards where replanting is no longer economically feasible several years post-planting [56]. In grapevines, recovery is linked to the absence of detectable phytoplasma in the tissues of recovered plants. Evidence suggests that the accumulation of hydrogen peroxide (H_2_O_2_) plays a key role, as indicated by the downregulation of scavenging enzymes such as catalase and ascorbate peroxidase scavenging enzymes, in recovered tissues, as observed in cv. Glera [34,56]. Additionally, Galetto et al. [79] provided the first evidence that *S. titanus* cannot acquire the phytoplasma from recovered grapevines. Their research also confirmed, in cv. Nebbiolo, the intracellular accumulation of H_2_O_2_, calcium, and callose in the phloem, along with reduced levels of antioxidant enzymes recovered from FD. For grapevine varieties with high recovery efficiency and stability, the practice of uprooting infected plants may not be necessary, as they no longer act as sources of inoculum. While recovered grapevines yield approximately 20% less than healthy plants, they produce significantly more than infected ones [80]. Consequently, maintaining infected plants to enable natural recovery appears to be a cost-effective and time-saving disease management strategy for growers [79], although it represents a legally prohibited practice in many contexts where the eradication of infected plants is mandatory [81]. Therefore, not taking into account territories subject to specific legislative restrictions for the containment of FD, for cultivars with an intermediate recovery potential, such as Chardonnay, the decision to maintain or replace infected plants depends on a combination of agronomic and economic factors, as well as the risk of new infections. Specifically, maintaining infected plants is more profitable when there is a lower yield per hectare. In contrast, replacing infected grapevines with new plants becomes relatively more advantageous under conditions such as longer productive lifespans, lower plant density per hectare, or an increase in grape market prices [56]. Also, field observations confirm that recovery from FD infection is highly dependent on the grapevine cultivar, with Barbera exhibiting a significantly higher recovery potential than Glera.

Previous research has demonstrated that recovery can be stimulated by abiotic stress, treatments with resistance inducers, antimicrobial compounds, and the application of mycorrhiza [36,56]. This line of research could be particularly promising, as it offers a more sustainable approach from both environmental and economic perspectives. The importance of understanding and enhancing recovery mechanisms is further highlighted by the significant economic impact of FD. For example, in 2005, in the Aleksandrovac region of Serbia, losses due to the destruction of vineyards by the FD, were estimated at around €3.2 million. In the same year, the Italian government and the EU provided €34 million in compensation for production losses and replanting [82]. In this regard, studies on FD-infected grapevine cultivars show that successful defense mechanisms are locally activated near symptomatic areas, involving jasmonate- and salicylate-mediated pathways, which contribute to compartmentalizing phytoplasma infections and limiting symptom spread [36]. Similarly, in BN-infected grapevines, treatments with resistance inducers, including benzothiadiazole, promoted recovery without negative effects on plant growth or yield [83]. These findings suggest that recovery, and the plant’s sensitivity to phytoplasma infection, are linked to a dynamic, locally regulated hormonal balance that varies among cell types and tissue compartments. Therefore, managing phytoplasma diseases may require strategies that support this hormonal regulation within specific tissues, allowing plants to effectively respond and recover. Further, the involvement of endophytes in the recovery process has been reported. The microbial community, in fact, associated with recovered plants, where phytoplasma replication is inactive, differs significantly from that of both healthy and diseased plants [30,84]. This suggests that the restructured microbial community in recovered plants may maintain its composition across seasons. Such findings point to a potential role for endophytes in protecting plants from re-infection. However, further studies are required to fully elucidate this role [84].

**Table 1:** Classification of grape cultivars according to their susceptibility. Grape susceptibility was classified into three levels based on symptom expression, phytoplasma load and localization, impact on yield, and recovery ability under natural infection pressure in vineyards.

Susceptibility to FD	Cultivar	Notes
High	Cortese (W) ^11^, Dolcetto (R) ^11^, Frankovka (R) ^5^, Garganega (W) ^7^, Istrian Malvasia (W) ^8^, Loureiro (W) ^10^, Perera (W) ^7^, Pinot Blanc (W) ^4^, Plovdina (R) ^5^, Ruché (R) ^11^, Sangiovese (R) ^7^, Vermentino (W) ^1^	High vulnerability to infection with low recovery ability.
Medium	Albarola (W) ^1^, Arneis (W) ^11^, Barbera (R) ^11^, Black Burgundy (R) ^5^, Cabernet Franc (R) ^6^, Cabernet Sauvignon (R) ^6^, Chardonnay (W) ^12^, Erbaluce (W) ^11^, Glera (W) ^7^, Grenache Noir (R) ^6^, Italian Riesling (W) ^5^, Pinot Gris (W) ^12^, Refosco D’Istria (R) ^7^, Riesling (W) ^7^, Sauvignon Blanc (W) ^6^, Smederevka (W) ^5^, Timorasso (W) ^11^, Trebbiano (W) ^9^, Zupski bojadiser (R) ^5^	Intermediate recovery ability; decision to maintain or replace depends on agronomic and economic factors.
Low	Brachetto (R) ^11^, Croatina (R) ^3^, Erbamat (W) ^2^, Freisa (R) ^11^, Magdaleine (W) ^6^, Merlot (R) ^6^, Moscato (W) ^12^, Nebbiolo (R) ^11^, Pinot Noir (R) ^6^, Syrah (R) ^6^, Teran (R) ^8^, Tocai Friulano (W) ^12^	Good recovery ability and tolerance; lower need for replacement.

The berry color for each cultivar will be distinguished as “(R)” for red and “(W)” for white. 1: [64]; 2: [74]; 3: [75]; 4: [85]; 5: [72]; 6: [78]; 7: [19]; 8: [77]; 9: [86]; 10: [53]; 11: [63]; 12: [6].

## 4. Important Local Cultivars That Have Received Little Attention

**Figure 2:**
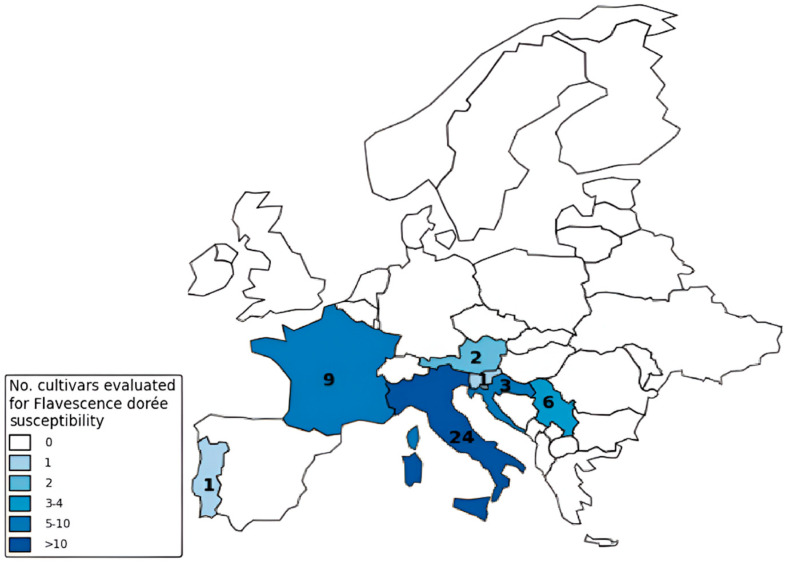
Distribution of grapevine cultivars evaluated for Flavescence dorée susceptibility in European countries (according to https://www.scopus.com/, accessed on 4 February 2025).

From what has been discussed above, it appears that there is a lot of information on some of international and non-international cultivars, analyzed in most cases within Europe (Figure 2). In this regard, it is useful to pay attention to all those cultivars that are only partially considered, with little or no scientific reference. In most cases these refer to areas where above all, the presence of other phytoplasmas is reported, and not necessarily that of FDp. This is the case, for example, reported by Davis et al. [13] in Israel and Greece, Pierro et al. [31] in China, or Abu Alloush et al. [21] in Jordan, where FDp is not present, leaving instead *Candidatus* phytoplasma solani as the most widespread. On the other hand, various studies have been conducted only in some European regions, such as the exhaustive overview offered by Northern Italy, highlighting differences in susceptibility between different local grapevine varieties [38,87]. For example, specific literature on Spanish cultivars is scarce. Autochthonous varieties such as Tempranillo, Garnacha, Albariño and Verdejo are widely cultivated in Spain, but information on their response to FD is limited or absent [18]. There is limited information regarding the vulnerability of key Austrian grape varieties too, such as Grüner Veltliner, Müller Thurgau, Blauer Zweigelt, and Blaufränkisch. Some varieties identified as susceptible hold significant importance in Austria, including Welschriesling and Rhine Riesling [88,89]. However, this figure is also a symptom of a reduced pressure and spread of the disease in these countries compared to other more affected ones, like Italy and France [25]. On the other hand, some countries, although with a limited diffusion, offer a more comprehensive overview of the spread of FD. This is the case of Portugal and the studies carried out especially on cv. Loureiro [90,91], of Slovenia on the cv. Modra frankinja [92,93], of Croatia with more specific studies on FDp infection on cvs. Plemenka Crvena, Istrian Malvasia and Pinot Gris [67,76,94], or of Serbia on the cv. Plovdina [72,95]. It is worth noting that in some neighboring countries, such as Hungary, and Bosnia and Herzegovina, research on FD is limited [96,97]. This is mainly because studies in these regions have focused on identifying other phytoplasmas (e.g., *Candidatus* phytoplasma solani), which have been detected in association with symptomatic grapevines [98,99]. The same applies to Montenegro [100] where however, Radonjić et al. [101] reported the first cases of FD in 2023. Additionally, many important regional, but widely used varieties may not have received as much research attention. This includes Sangiovese in Italy, Tempranillo in Spain, or Zinfandel in California, which are among the main regional grape cultivars [102,103]. Moreover, certain newer hybrid cultivars might also lack comprehensive data on their susceptibility to FD, particularly those being tested for climate adaptability or disease resistance. In this regard, interspecific viticultural hybrids are acquiring a strategic role in genetic improvement: several recent studies indicate that cultivars obtained from crosses with species such as *Vitis amurensis* or *V. labrusca* may show a lower susceptibility to FD infection or present attenuated symptoms. To support these observations, several breeding programs, such as the BIORES project, are selecting tolerant genotypes to be used either directly or as a genetic source for the introduction of resistance traits by conventional crossing or genome editing techniques [104]. The use of rootstocks derived from resistant species has also shown promise, as it can help limit the multiplication and systemic spread of phytoplasma in grafted plants [105].

Market demands often affect the wine industry, and research funding tends to prioritize popular grape varieties [106]. For instance, well-known varieties like Pinot Noir or Merlot attract more scientific focus due to their widespread use and economic significance [103]. Moreover, there is no unified, standardized method for assessing grapevine susceptibility to FD, which can result in inconsistent or incomplete data. Some studies may focus on the disease vector or environmental factors instead of directly evaluating susceptibility in different cultivars [85,107]. Further, how researchers define susceptibility, whether through symptom expression or disease progression, can differ, making it challenging to compare findings across studies.

In conclusion, the difficulty in gathering comprehensive information on the susceptibility of certain grapevine cultivars to FD is due to a range of interconnected factors. These include the emphasis on commercially significant cultivars, inconsistent research methodologies, legislative restrictions, and the complex biological and environmental interactions at play. This underscores the need for further research to fill these gaps and develop more effective management strategies.

## 5. Innovative Strategies for Management of Grapevine Yellows and Addressing Susceptibility

The GY susceptibility of several major grapevine varieties poses a serious problem in viticulture, making traditional control measures insufficient to protect them. Therefore, innovative strategies for grapevine disease management are essential to address this issue [108,109].

Rapid and non-destructive methods of GY detection in vineyards represent innovations that could effectively contrast the GY spread and have gained significant attention in recent years [110]. These new technologies, based on optical sensors, capture alterations in leaf optical properties and have already shown the ability to distinguish between healthy and GY-infected plants by analyzing plant reflectance in the field [111]. Considering FD, the methods applied so far include RGB imaging [112], multispectral imaging [113,114], and hyperspectral data [115,116]. This last appears to be the most promising due to its wide range of potential applications, extending beyond rapid detection alone. Recently, the potential of hyperspectral data to distinguish between FD-positive and FD-negative grapevines in asymptomatic plants before symptoms appear was highlighted [24]. Moreover, Oerke et al. [117,118] also emphasized the potential application of hyperspectral data in research focused on the susceptibility of varieties to diseases, as demonstrated for *Plasmopara viticola*. However, despite these intriguing results, no research has yet specifically evaluated for GY across different varieties, representing a significant opportunity for future studies.

Endophytic communities (i.e., bacteria and fungi) play a crucial role in plant health by enhancing stress tolerance, promoting growth, and protecting against pathogens through the production of bioactive compounds and competition with harmful microbes [119,120,121]. Some evidence suggest that endophytic bacteria may contribute to the recovery phenomenon in grapevines affected by GY [30,122], and also endophytic fungi can potentially have some role in this phenomenon [123,124]. Indeed, Bulgari et al. [84,125] demonstrated that the diversity of the grapevine endophytic bacterial community is greater in recovered grapevines previously affected by FD or BN than in diseased or healthy plants. Moreover, beneficial endophytic bacteria, such as *Pseudomonas migulae*, have shown potential effects in inducing systemic resistance to FDp in the experimental host *Catharanthus roseus* [126]. Thus, exploring the dynamics and potential applications of endophytic communities could offer a promising strategy to enhance grapevine resistance or tolerance to FD, even in varieties that are naturally susceptible. However, additional research is needed before these approaches can be effectively implemented to mitigate GY at a field scale.

Other promising innovative techniques include genetic approaches, which represent a powerful tool for addressing grapevine diseases. These approaches aid in the selection of new grapevine varieties capable of reducing or eliminating the need for agrochemical inputs while promoting ecological and sustainable management [127]. In some cases, these techniques have been authorized for use in breeding, whereas in others, strict regulatory frameworks, such as those in the European Union, have been applied [128]. A key example is marker-assisted selection (MAS) and the Clustered Regularly Interspaced Short Palindromic Repeats/CRISPR-associated protein 9 (CRISPR/Cas9) system, which facilitate the detection of useful alleles in the plant genome for resistance or tolerance and provide precise tools for modifying genes associated with susceptibility to plant diseases, respectively [129,130]. Both techniques have already been successfully applied to identify or modify genes of interest for resistance against the most damaging pathogens affecting grapevine, including *Plasmopara viticola*, *Erysiphe necator*, *Botrytis cinerea* and *Guignardia bidwellii*, as well *Daktulosphaira vitifoliae* [131,132,133,134,135,136,137,138]. However, these techniques have yet to be applied to develop grapevine varieties resistant or tolerant to GY, including FD, highlighting a significant gap in this research area.

## 6. Conclusions

There is currently substantial and insightful knowledge available regarding the disease dynamics of FD. Phytoplasma’s nature, life cycle, and the behavior of its specific insect vector have become increasingly well understood. As a result, every stage and consequence of the interaction between the phytoplasma and its host, as well as the symptoms that occur once the pathogenic relationship with the vine is established, begin to be examined and described more accurately. Furthermore, research has shed light on the feeding behavior of the vector, which appears to vary depending on the grapevine cultivar, thereby influencing, either positively or negatively, the efficiency of phytoplasma acquisition and its subsequent spread within the plant. In this regard, the recovery phenomenon also remains highly cultivar-dependent and should be further investigated in order to determine its causes. However, current studies remain largely concentrated in regions experiencing exceptionally high FD pressure, leading to a focus on a limited number of cultivars that are often specific to a single geographical area. This is not merely a matter of examining vector behavior or the general susceptibility of a cultivar, but instead of conducting studies on individual cultivars in regions beyond the countries where FD is most prevalent. As previously noted, only a handful of countries, despite experiencing low FD pressure, have not only investigated and confirmed the presence of the phytoplasma and its vector across multiple national wine-growing areas, but have also specifically analyzed their local cultivars.

In most cases, FD has only been detected when the risk of its spread became apparent, either through vector capture or the emergence of symptomatic plants. Despite each country’s annual implementation of control measures, FD continues to advance across Europe, sparing no neighboring state. This underscores the importance of maintaining proactive research and surveillance in regions where the disease remains scarce while intensifying in-depth investigations into its dynamics in areas where it is already established, particularly in relation to the susceptibility of local cultivars. The ultimate objective is to develop a comprehensive understanding of the disease, its variability across different regions, and its interactions with various cultivars. Moreover, integrating innovative strategies, such as deploying optical sensors, fostering beneficial interactions within endophytic communities, employing MAS, and advancing genome editing techniques like the CRISPR/Cas9 system, could provide a comprehensive and sustainable approach to managing GY/FD, addressing the susceptibility of grapevines, and enhancing disease resistance or tolerance. Such insights will facilitate the development of more effective containment strategies and support the identification and promotion of tolerant or resistant cultivars, fostering further research into sustainable long-term solutions.

## Data Availability

Not applicable.

## Abbreviations

The following abbreviations are used in this manuscript:

FD	Flavescence dorée
FDp	Flavescence dorée phytoplasma
BN	Bois Noir
GY	Grapevine Yellows
ROS	Reactive Oxygen Species
TCA	Tricarboxylic acid
IAA	Auxin-Indole-3-Acetic Acid
PAMPs	Pathogen-Associated Molecular Patterns
DAMPs	Damage-Associated Molecular Patterns
ETI	Effector-Triggered Immunity
JA	Jasmonic Acid
SA	Salicylic Acid
NGS	Next-generation Sequencing
ELISA	Enzyme-Linked Immunosorbent Assay
PCR	Polymerase Chain Reaction
qPCR	Real time Quantitative Polymerase Chain Reaction
dPCR	Digital Polymerase Chain Reaction
ddPCR	Droplet Digital Polymerase Chain Reaction
LAMP	Loop-Mediated Isothermal Amplification
MAS	Marker-Assisted Selection
CRISPR/Cas9	Clustered Regularly Interspaced Short Palindromic Repeats/CRISPR-associated protein 9

## References

[1] International Organisation of Vine and Wine (OIV) (2024). State of the World Vine and Wine Sector 2023.

[2] Margaria P., Palmano S. (2011). Response of the *Vitis vinifera* L. Cv. “Nebbiolo” Proteome to Flavescence Dorée Phytoplasma Infection. Proteomics.

[3] Angelini E., Constable F., Duduk B., Fiore N., Quaglino F., Bertaccini A. (2018). Grapevine Phytoplasmas. Phytoplasmas: Plant Pathogenic Bacteria—I: Characterisation and Epidemiology of Phytoplasma—Associated Diseases.

[4] Bernardini C., Santi S., Mian G., Levy A., Buoso S., Suh J.H., Wang Y., Vincent C., van Bel A.J.E., Musetti R. (2022). Increased susceptibility to Chrysanthemum Yellows phytoplasma infection in Atcals7ko plants is accompanied by enhanced expression of carbohydrate transporters. Planta.

[5] Bertaccini A., Duduk B., Paltrinieri S., Contaldo N. (2014). Phytoplasmas and Phytoplasma Diseases: A Severe Threat to Agriculture. Am. J. Plant Sci..

[6] Kube M., Schneider B., Kuhl H., Dandekar T., Heitmann K., Migdoll A.M., Reinhardt R., Seemüller E. (2008). The linear chromosome of the plant-pathogenic mycoplasma ‘*Candidatus* Phytoplasma mali’. BMC Genom..

[7] Foissac X., Wilson M.R., Weintraub P.G., Jones P. (2009). Current and possible future distributions of phytoplasma diseases and their vectors. Phytoplasmas: Genomes, Plant Hosts and Vectors.

[8] Weintraub P.G., Beanland L. (2006). Insect vectors of phytoplasmas. Annu. Rev. Entomol..

[9] Bertaccini A. (2007). Phytoplasmas: Diversity, Taxonomy, and Epidemiology. Front. Biosci..

[10] Canel A., Zambon Y., Bertaccini A., Paltrinieri S., Contaldo N. (2014). Flavescenza Dorata Della Vite Sotto Controllo Nel Trevigiano. L’Informatore Agrar..

[11] Wang R., Bai B., Li D., Wang J., Huang W., Wu Y., Zhao L. (2024). Phytoplasma: A plant pathogen that cannot be ignored in agricultural production—Research progress and outlook. Mol. Plant Pathol..

[12] Debonneville C., Mandelli L., Brodard J., Groux R., Roquis D., Schumpp O. (2022). The Complete Genome of the “Flavescence dorée” Phytoplasma Reveals Characteristics of Low Genome Plasticity. Biology.

[13] Davis R.E., Dally E.L., Tanne E., Rumbos I.C. (1997). Phytoplasmas associated with grapevine yellows in Israel and Greece belong to the stolbur Phytoplasma subgroup, 16SrXII-A. J. Plant Pathol..

[14] Rao G.P., Fiore N., Bertaccini A., Liefting L.W. (2018). Phytoplasmas: An Update. Phytoplasmas: Plant Pathogenic Bacteria—I: Characterisation and Epidemiology of Phytoplasma—Associated Diseases.

[15] Firrao G., Andersen M.T., Bertaccini A., Boudon-Padieu E., Bové J., Daire X., Davis R.E., Fletcher J., Garnier M., Gibb K.S. (2004). ‘*Candidatus* Phytoplasma’, a taxon for the wall-less, non-helical prokaryotes that colonize plant phloem and insects. Int. J. Syst. Evol. Microbiol..

[16] Quaglino F., Zhao Y., Casati P., Bulgari D., Bianco P.A., Wei W., Davis R.E. (2013). ‘*Candidatus* Phytoplasma solani’, a novel taxon associated with stolbur-and bois noir-related diseases of plants. Int. J. Syst. Evol. Microbiol..

[17] EPPO Grapevine Flavescence Dorée Phytoplasma (PHYP64). https://gd.eppo.int/taxon/PHYP64.

[18] Batlle A., Angeles Martínez M., Laviña A. (2000). Occurrence, Distribution and Epidemiology of Grapevine Yellows in Spain. Europ. J. Plant Pathol..

[19] Constable F., Bertaccini A. (2017). Worldwide Distribution and Identification of Grapevine Yellows Diseases. Grapevine Yellows Diseases and Their Phytoplasma Agents.

[20] Davis R.E., Dally E.L., Zhao Y., Wolf T.K. (2018). Genotyping Points to Divergent Evolution of *‘Candidatus* Phytoplasma Asteris’ Strains Causing North American Grapevine Yellows and Strains Causing Aster Yellows. Plant Dis..

[21] Abu Alloush A.H., Bianco P.A., Busato E., Alkhawaldeh Y., Alma A., Tedeschi R., Quaglino F. (2023). Grapevine Yellows in Jordan: Associated Phytoplasmas, Putative Insect Vectors and Reservoir Plants. Plant Pathol..

[22] Belli G., Bianco P.A., Conti M. (2010). Grapevine Yellows in Italy: Past, present and future. J. Plant Pathol..

[23] Arnaud G., Malembic-Maher S., Salar P., Bonnet P., Maixner M., Marcone C., Boudon-Padieu E., Foissac X. (2007). Multilocus Sequence Typing Confirms the Close Genetic Interrelatedness of Three Distinct Flavescence Doreé Phytoplasma Strain Clusters and Group 16SrV Phytoplasmas Infecting Grapevine and Alder in Europe. Appl. Environ. Microbiol..

[24] Pedrelli A., Carli M., Panattoni A., Pellegrini E., Rizzo D., Nali C., Cotrozzi L. (2024). Investigating a New Alarming Outbreak of Flavescence Dorée in Tuscany (Central Italy): Molecular Characterization and Map Gene Typing Elucidate the Complex Phytoplasma Ecology in the Vineyard Agroecosystem. Front. Plant Sci..

[25] Malembic-Maher S., Desqué D., Khalil D., Salar P., Bergey B., Danet J.L., Duret S., Dubrana-Ourabah M.P., Beven L., Ember I. (2020). When a Palearctic Bacterium Meets a Nearctic Insect Vector: Genetic and Ecological Insights into the Emergence of the Grapevine Flavescence Dorée Epidemics in Europe. PLoS Pathog..

[26] Boudon-Padieu E. (2002). Flavescence doree of the grapevine. Knowledge and new developments in epidemiology-aetiology and diagnosis [*Vitis vinifera* L.]. Atti Delle Giornate Fitopatol..

[27] Filippin L., Jovic’ J., Cvrkovic’ T., Forte V., Clair D., Tosevski I., Boudon-Padieu E., Borgo M., Angelini E. (2009). Molecular characteristics of phytoplasmas associated with Flavescence doreé in clematis and grapevine and preliminary results on the role of *Dictyophara europaea* as a vector. Plant Pathol..

[28] Caudwell A. (1957). Deux années d’étude sur la Flavescence dorée, nouvelle maladie grave de la vigne. Ann. Amélior. Plantes.

[29] Caudwell A. (1990). Epidemiology and Characterization of Flavescence Dorée (FD) and Other Grapevine Yellows. Agronomie.

[30] Oliveira M.J.R.A., Roriz M., Vasconcelos M.W., Bertaccini A., Carvalho S.M.P. (2019). Conventional and Novel Approaches for Managing “Flavescence Dorée” in Grapevine: Knowledge Gaps and Future Prospects. Plant Pathol..

[31] Pierro R., Semeraro T., Luvisi A., Garg H., Vergine M., De Bellis L., Gill H.K. (2019). The Distribution of Phytoplasmas in South and East Asia: An Emerging Threat to Grapevine Cultivation. Front. Plant Sci..

[32] Bigozzi M. (2023). Indagine Sul Ruolo Di *Orientus ishidae* (Matsumura, 1902) Nella Diffusione Della Flavescenza Dorata Della Vite in Piemonte. Master’s Thesis.

[33] Dermastia M. (2019). Plant Hormones in Phytoplasma Infected Plants. Front. Plant Sci..

[34] Margaria P., Abbà S., Palmano S. (2013). Novel Aspects of Grapevine Response to Phytoplasma Infection Investigated by a Proteomic and Phospho-Proteomic Approach with Data Integration into Functional Networks. BMC Genom..

[35] Coppari F. (2019). Flavescenza Dorata e Sue Implicazioni Nel Panorama Viticolo. Bachelor’s Thesis.

[36] Casarin S., Vincenzi S., Esposito A., Filippin L., Forte V., Angelini E., Bertazzon N. (2023). A successful defence strategy in grapevine cultivar ‘Tocai friulano’ provides compartmentation of grapevine Flavescence dorée phytoplasma. BMC Plant Biol..

[37] Huancas F., Coronel A., Vidal R., Berres S., Brito H. (2024). A Mathematical Model of Flavescence Dorée in Grapevines by Considering Seasonality. Math. Biosci. Eng..

[38] Rigamonti I.E., Salvetti M., Girgenti P., Bianco P.A., Quaglino F. (2023). Investigation on Flavescence Dorée in North-Western Italy Identifies Map-M54 (16SrV-D/Map-FD2) as the Only Phytoplasma Genotype in *Vitis vinifera* L. and Reveals the Presence of New Putative Reservoir Plants. Biology.

[39] Pearson R.C., Pool R.M., Gonsalves D., Goffinet M.C. (1985). Occurrence of Flavescence Dorée-like Symptoms on “White Riesling” Grape Vines in New York, U.S.A. Phytopathol. Mediterr..

[40] Seemüller E., Garnier M., Schneider B., Razin S., Herrmann R. (2002). Mycoplasmas of Plants and Insects. Molecular Biology and Pathogenicity of Mycoplasmas.

[41] Haider M.W., Sharma A., Majumdar A., Fayaz F., Bromand F., Rani U., Singh V.K., Saharan M.S., Tiwari R.K., Lal M.K. (2024). Unveiling the Phloem: A Battleground for Plant Pathogens. Phytopathol. Res..

[42] van Bel A.J.E. (2019). Sieve Elements: The Favourite Habitat of Phytoplasmas. Methods in Molecular Biology.

[43] Trivellone V., Dietrich C.H. (2021). Evolutionary Diversification in Insect Vector-Phytoplasma-Plant Associations. Ann. Entomol. Soc. Am..

[44] Van Bel A.J.E., Musetti R. (2019). Sieve Element Biology Provides Leads for Research on Phytoplasma Lifestyle in Plant Hosts. J. Exp. Bot..

[45] Christensen N.M., Axelsen K.B., Nicolaisen M., Schulz A. (2005). Phytoplasmas and Their Interactions with Hosts. Trends Plant Sci..

[46] Hogenhout S.A., Oshima K., Ammar E.D., Kakizawa S., Kingdom H.N., Namba S. (2008). Phytoplasmas: Bacteria that manipulate plants and insects. Mol. Plant Pathol..

[47] Marcone C. (2014). Molecular Biology and Pathogenicity of Phytoplasmas. Ann. Appl. Biol..

[48] Buoso S., Pagliari L., Musetti R., Martini M., Marroni F., Schmidt W., Santi S. (2019). “*Candidatus* Phytoplasma Solani” Interferes with the Distribution and Uptake of Iron in Tomato. BMC Genom..

[49] Hasanuzzaman M., Nahar K., Anee T.I., Fujita M. (2017). Glutathione in Plants: Biosynthesis and Physiological Role in Environmental Stress Tolerance. Physiol. Mol. Biol. Plants.

[50] dos Santos C., Franco O.L. (2023). Pathogenesis-Related Proteins (PRs) with Enzyme Activity Activating Plant Defense Responses. Plants.

[51] Hren M., Ravnikar M., Brzin J., Ermacora P., Carraro L., Bianco P.A., Casati P., Borgo M., Angelini E., Rotter A. (2009). Induced Expression of Sucrose Synthase and Alcohol Dehydrogenase I Genes in Phytoplasma-Infected Grapevine Plants Grown in the Field. Plant Pathol..

[52] Rojas C.M., Senthil-Kumar M., Tzin V., Mysore K.S. (2014). Regulation of Primary Plant Metabolism during Plant-Pathogen Interactions and Its Contribution to Plant Defense. Front. Plant Sci..

[53] Oliveira M.J.R.A., Castro S., Paltrinieri S., Bertaccini A., Sottomayor M., Santos C.S., Vasconcelos M.W., Carvalho S.M.P. (2020). “Flavescence Dorée” Impacts Growth, Productivity and Ultrastructure of *Vitis vinifera* Plants in Portuguese “Vinhos Verdes” Region. Sci. Hortic..

[54] Miura C., Sugawara K., Neriya Y., Minato N., Keima T., Himeno M., Maejima K., Komatsu K., Yamaji Y., Oshima K. (2012). Functional Characterization and Gene Expression Profiling of Superoxide Dismutase from Plant Pathogenic Phytoplasma. Gene.

[55] Hoshi A., Oshima K., Kakizawa S., Ishii Y., Ozeki J., Hashimoto M., Komatsu K., Kagiwada S., Yamaji Y., Namba S. (2009). A unique virulence factor for proliferation and dwarfism in plants identified from a phytopathogenic bacterium. Proc. Natl. Acad. Sci. USA.

[56] Ripamonti M., Pacifico D., Roggia C., Palmano S., Rossi M., Bodino N., Marzachì C., Bosco D., Galetto L. (2020). Recovery from Grapevine Flavescence Dorée in Areas of High Infection Pressure. Agronomy.

[57] He J., Li R., Xu C., Chen X., Yao J., Li Z., Cheng Y. (2025). Enhancing Fruit Resistance against Fungal Pathogens Using a Pathogen-Associated Molecular Pattern PdEIX. J. Agric. Food Chem..

[58] Harris F.M., Mou Z. (2024). Damage-Associated Molecular Patterns and Systemic Signaling. Phytopathology.

[59] Carreón-Anguiano K.G., Vila-Luna S.E., Sáenz-Carbonell L., Canto-Canché B. (2023). Novel Insights into Phytoplasma Effectors. Horticulturae.

[60] Nguyen Q.M., Iswanto A.B.B., Son G.H., Kim S.H. (2021). Recent Advances in Effector-Triggered Immunity in Plants: New Pieces in the Puzzle Create a Different Paradigm. Int. J. Mol. Sci..

[61] Gazzetta Ufficiale dell’Unione Europea (2016). Parlamento Europeo e Il Consiglio Dell’Unione Europea. Regolamento (UE) 2016/2031 del Parlamento Europeo E del Consiglio.

[62] Tessitori M., La Rosa R., Marzachì C. (2018). Flavescence Dorée and Bois Noir Diseases of Grapevine Are Evolving Pathosystems. Plant Health Prog..

[63] Ripamonti M., Pegoraro M., Morabito C., Gribaudo I., Schubert A., Bosco D., Marzachì C. (2020). Susceptibility to Flavescence Dorée of Different *Vitis vinifera* Genotypes from North-Western Italy. Plant Pathol..

[64] Boselli M. (1999). Spatial Distribution and Severity of Grapevine Yellows on Albarola and Vermentino Grapevine (*Vitis vinifera* L.) Cultivars in Eastern Liguria (Northern Italy). Adv. Hort. Sci..

[65] Morabito C., Pagliarani C., Lovisolo C., Ripamonti M., Bosco D., Marzachì C., Roitsch T., Schubert A., Lunn J. (2024). The sucrose signalling route controls Flavescence dorée phytoplasma load in grapevine leaves. J. Exp. Bot..

[66] Roggia C., Caciagli P., Galetto L., Pacifico D., Veratti F., Bosco D., Marzachì C. (2014). Flavescence Dorée Phytoplasma Titre in Field-Infected Barbera and Nebbiolo Grapevines. Plant Pathol..

[67] Davosir D., Šola I., Šeruga Musić M. (2024). Physiological Responses of Grapevine (*Vitis vinifera* Var. ‘Pinot Gris’) Affected by Different Flavescence Dorée Genotypes: Dynamics through the Development of Phytoplasma Infection. J. Plant Dis. Prot..

[68] Mir Z.A., Ali S., Manzoor S., Sharma D., Sharma D., Tyagi A., Wani A.W., Kumar S., Ayele B.T. (2025). Plant Defense Hormones: Thermoregulation and Their Role in Plant Adaptive Immunity. J. Plant Growth Regul..

[69] Paolacci A.R., Catarcione G., Ederli L., Zadra C., Pasqualini S., Badiani M., Musetti R., Santi S., Ciaffi M. (2017). Jasmonate-mediated defence responses, unlike salicylate-mediated responses, are involved in the recovery of grapevine from bois noir disease. BMC Plant Biol..

[70] Schneider A., Boccacci P., Botta R. (2003). Genetic Relationships among Grape Cultivars from North-Western Italy. Acta Hortic..

[71] Galetto L., Miliordos D.E., Pegoraro M., Sacco D., Veratti F., Marzachì C., Bosco D. (2016). Acquisition of Flavescence Dorée Phytoplasma by *Scaphoideus titanus* Ball from Different Grapevine Varieties. Int. J. Mol. Sci..

[72] Kuzmanovic S., Josic D., Starovic M., Ivanovic Z., Popovic T., Trkulja N., Bajic-Raymond S., Stojanovic S. (2011). Detection of Flavescence Dorée Phytoplasma Strain C on Different Grapevine Cultivars in Serbian Vineyards. Bulg. J. Agric. Sci..

[73] Ripamonti M., Galetto L., Maron F., Marzachì C., Bosco D. (2022). *Scaphoideus titanus* Fitness on Grapevine Varieties with Different Susceptibility to Flavescence Dorée Phytoplasma. J. Appl. Entomol..

[74] Belli G., Bianco A., Casati P., Scattini G. (2000). Serious and widespread outbreaks of Flavescence dorée in vines in Lombardy. L’Informatore Agrar..

[75] Vercesi A., Scattini G. (2000). Spread of flavescencedorée of grapes in the Oltrepo Pavese in 1999. Vignevini.

[76] Martelli G.P., Boudon-Padieu E., Martelli G.P., Boudon-Padieu E. (2006). Directory of Infectious Diseases of Grapevines and Viroses and Virus-like Diseases of the Grapevine: Bibliographic Report 1998–2004.

[77] Plavec J., Budinšćak, Križanac I., Škorić D., Foissac X., ŠerugaMusić M. (2019). Multilocus Sequence Typing Reveals the Presence of Three Distinct Flavescence Dorée Phytoplasma Genetic Clusters in CroatianVineyards. Plant Pathol..

[78] Eveillard S., Jollard C., Labroussaa F., Khalil D., Perrin M., Desqué D., Salar P., Razan F., Hévin C., Bordenave L. (2016). Contrasting Susceptibilities to Flavescence Dorée in *Vitis vinifera*, Rootstocks and Wild Vitis Species. Front. Plant Sci..

[79] Galetto L., Miliordos D., Roggia C., Rashidi M., Sacco D., Marzachì C., Bosco D. (2014). Acquisition Capability of the Grapevine Flavescence Dorée by the Leafhopper Vector *Scaphoideus titanus* Ball CorrelateswithPhytoplasma Titre in the Source Plant. J. Pest Sci..

[80] Morone C., Boveri M., Giosuè S., Gotta P., Rossi V., Scapin I., Marzachi C. (2007). Epidemiology of Flavescence Dorée in Vineyards in Northwestern Italy. Phytopathology.

[81] Parlamento Europeo e Il Consiglio Dell’Unione Europea (2022). Gazzetta Ufficiale Dell’unione Europea. L.A.C. Regolamento di Esecuzione (UE) 2022/1630 Della Commissione.

[82] Chuche J., Thiéry D. (2014). Biology and Ecology of the Flavescence Dorée Vector *Scaphoideus titanus:* A Review. Agron. Sustain. Dev..

[83] Romanazzi G., Murolo S., Feliziani E. (2013). Effects of an Innovative Strategy to Contain Grapevine Bois Noir: Field Treatment with Resistance Inducers. Am. Phytopathol. Soc..

[84] Bulgari D., Casati P., Quaglino F., Bianco P.A. (2014). Endophytic Bacterial Community of Grapevine Leaves Influenced by Sampling Date and Phytoplasma Infection Process. BMC Microbiol..

[85] Bressan A., Spiazzi S., Girolami V., Boudon-Padieu E. (2005). Acquisition Efficiency of Flavescence Dorée Phytoplasma by *Scaphoideus titanus* Ball from Infected Tolerant or Susceptible Grapevine Cultivars or Experimental Host Plants. Vitis.

[86] Boulent J., St-Charles P.L., Foucher S., Théau J. (2020). Automatic Detection of Flavescence Dorée Symptoms Across White Grapevine Varieties Using Deep Learning. Front. Artif. Intell..

[87] Ripamonti M., Maron F., Cornara D., Marzachì C., Fereres A., Bosco D. (2021). *Scaphoideus titanus* Ball Feeding Behaviour on Three Grapevine Cultivars with Different Susceptibilities to Flavescence Dorée. J. Insect Physiol..

[88] Steffek R., Reisenzein H., Zeisner N. (2007). Analysis of the Pest Risk from Grapevine Flavescence Dorée Phytoplasma to Austrian Viticulture. EPPO Bull..

[89] Reisenzein H., Steffek R. (2011). First Outbreaks of Grapevine “Flavescence Dorée” in Austrian Viticulture. Bull. Insectology.

[90] de Sousa E., Casati P., Cardoso F., Baltazar C., Durante G., Quaglino F., Bianco P.A. (2010). Flavescence dorée phytoplasma affecting grapevine (*Vitis vinifera*) newly reported in Portugal. Plant Pathol..

[91] Teixeira A., Martins V., Frusciante S., Cruz T., Noronha H., Diretto G., Gerós H. (2020). Flavescence Dorée-Derived Leaf Yellowing in Grapevine (*Vitis vinifera* L.) Is Associated to a General Repression of Isoprenoid Biosynthetic Pathways. Front. Plant Sci..

[92] Prezelj N., Nikolić P., Gruden K., Ravnikar M., Dermastia M. (2013). Spatiotemporal Distribution of Flavescence Dorée Phytoplasma in Grapevine. Plant Pathol..

[93] Prezelj N., Covington E., Roitsch T., Gruden K., Fragner L., Weckwerth W., Chersicola M., Vodopivec M., Dermastia M. (2016). Metabolic Consequences of Infection of Grapevine (*Vitis vinifera* L.) cv. “ModraFrankinja” with Flavescence Dorée Phytoplasma. Front. Plant Sci..

[94] Musić M.Š., Škoriæ D., Haluška I., Križanac I., Plavec J., Mikec I. (2011). First Report of Flavescence Dorée-Related Phytoplasma Affecting Grapevines in Croatia. Plant Dis..

[95] Kuzmanović S., Jošić D., Ivanović Ž., Popović T., Stojanović S., Aleksić G., Starović M. (2011). A Study of Suitability of Grapevine Cultivar Plovdina as a Possible Indicator Plant for Flavescence Dorée Disease. Afr. J. Agric. Res..

[96] Radulović M., Hrnčić S., Đurić G., Delić D. Results of the surveillance for “flavescence dorée” phytoplasma and *Scaphoideus titanus* in the Republic of Srpska (Bosnia and Herzegovina). Proceedings of the 19th Congress of ICVGS.

[97] Szamatari E., Merkely B., Végh A. (2024). Infection of ‘*Candidatus* Phytoplasma Vitis’ in a Hungarian Vineyard East of the Danube. Georg. Agric..

[98] Kuzmanovic S., Martinizi M., Ermacora P., Ferrini F., Starovic M., Tosic M., Carraro L., Osler R. (2008). Incidence and molecular characterization of flavescence dorée and stolbur phytoplasmas in grapevine cultivars from different viticultural areas of Serbia. Vitis.

[99] Ember I., Bodor P., Zsófi Z., Pálfi Z., Ladányi M., Pásti G., Deák T., Nyitrainé D.S., Bálo B., Szekeres A. (2018). Bois Noir Affects the Yield and Wine Quality of *Vitis vinifera* L. cv. ‘Chardonnay’. Eur. J. Plant Pathol..

[100] Kosovac A., Radonjić S., Hrnčić S., Krstić O., Toševski I., Jović J. (2016). Molecular Tracing of the Transmission Routes of Bois Noir in Mediterranean Vineyards of Montenegro and Experimental Evidence for the Epidemiological Role of *Vitex Agnus-Castus* (Lamiaceae) and Associated *Hyalesthes obsoletus* (Cixiidae). Plant Pathol..

[101] Radonjić S., Krstić O., Cvrković T., Hrnčić S., Marinković S., Mitrović M., Toševski I., Jović J. (2023). The First Report on the Occurrence of Flavescence Dorée Phytoplasma Affecting Grapevine in Vineyards of Montenegro and an Overview of Epidemic Genotypes in Natural Plant Reservoirs. J. Plant Pathol..

[102] Zombardo A., Meneghetti S., Morreale G., Calò A., Costacurta A., Storchi P. (2022). Study of Inter-and Intra-Varietal Genetic Variability in Grapevine Cultivars. Plants.

[103] OIV (2017). Distribution of the World’s Grapevine Varieties.

[104] Cavagna F., Guerrieri E., Danzi D., Palmano S., Marzachi C., Mori N., Polverari A. Exploring diversity of grapevine responses to Flavescence dorée infection. Proceedings of the Open International Conference on Grapevine Physiology and Biotechnology (Open-GPB2024).

[105] Jeger M., Bragard C., Caffier D., Candresse T., Chatzivassiliou E., Dehnen-Schmutz K., Gilioli G., Jaques Miret J.A., Macleod A., Navarro M.N. (2016). Risk to Plant Health of Flavescence Dorée for the EU Territory. EFSA J..

[106] Mueller-Loose S., Rey Del R., Marinelli N., Pomarici E., Contini C. (2021). State of the International Wine Markets in 2023. The wine market at a crossroads: Temporary or structural challenges. Wine Economics and Policy.

[107] Steffek R., Reisenzein H., Strauss G., Leichtfried T., Hofrichter J., Kopacka I., Schwarz M., Pusterhofer J., Biedermann R., Renner W. (2011). VitisCLIM, a Project Modelling Epidemiology and Economic Impact of Grapevine “Flavescence dorée” Phytoplasma in Austrian Viticulture under a Climate Change Scenario. Bull. Insectology.

[108] Butiuc-Keul A., Coste A. (2023). Biotechnologies and Strategies for Grapevine Improvement. Horticulturae.

[109] Ferro M.V., Catania P. (2023). Technologies and Innovative Methods for Precision Viticulture: A Comprehensive Review. Horticulturae.

[110] Cotrozzi L., Couture J.J. (2020). Hyperspectral Assessment of Plant Responses to Multi-Stress Environments: Prospects for Managing Protected Agrosystems. Plants People Planet.

[111] Bendel N., Backhaus A., Kicherer A., Köckerling J., Maixner M., Jarausch B., Biancu S., Klück H.C., Seiffert U., Voegele R.T. (2020). Detection of Two Different Grapevine Yellows in *Vitis vinifera* Using Hyperspectral Imaging. Remote Sens..

[112] Tardif M., Amri A., Keresztes B., Deshayes A., Martin D., Greven M., Da Costa J.P. (2022). Two-Stage Automatic Diagnosis of Flavescence dorée Based on Proximal Imaging and Artificial Intelligence: A Multi-Year and Multi-Variety Experimental Study. Oeno One.

[113] Barjaktarović M., Santoni M., Faralli M., Bertamini M., and Bruzzone L. A multispectral acquisition system for potential detection of Flavescence dorée. Proceedings of the 2022 30th Telecommunications Forum (TELFOR).

[114] Daglio G., Cesaro P., Todeschini V., Lingua G., Lazzari M., Berta G., Massa N. (2022). Potential Field Detection of Flavescence dorée and Esca Diseases Using a Ground Sensing Optical System. Biosyst. Eng..

[115] Mas Garcia S., Ryckewaert M., Abdelghafour F., Metz M., Moura D., Feilhes C., Prezman F., Bendoula R. (2021). Combination of Multivariate Curve Resolution with Factorial Discriminant Analysis for the Detection of Grapevine Diseases Using Hyperspectral Imaging. A Case Study: Flavescence Dorée. Analyst.

[116] Imran H.A., Zeggada A., Ianniello I., Melgani F., Polverari A., Baroni A., Danzi D., Goller R. (2023). Low-Cost Handheld Spectrometry for Detecting Flavescence dorée in Vineyards. Appl. Sci..

[117] Oerke E.C., Herzog K., Toepfer R. (2016). Hyperspectral Phenotyping of the Reaction of Grapevine Genotypes to *Plasmopara viticola*. J. Exp. Bot..

[118] Oerke E.C., Juraschek L., Steiner U. (2023). Hyperspectral Mapping of the Response of Grapevine Cultivars to *Plasmopara viticola* Infection at the Tissue Scale. J. Exp. Bot..

[119] Rodriguez R.J., White J.F., Arnold A.E., Redman R.S. (2009). Fungal Endophytes: Diversity and Functional Roles: Tansley Review. New Phytol..

[120] Santoyo G., Moreno-Hagelsieb G., del Carmen Orozco-Mosqueda M., Glick B.R. (2016). Plant Growth-Promoting Bacterial Endophytes. Microbiol. Res..

[121] Vergine M., Meyer J.B., Cardinale M., Sabella E., Hartmann M., Cherubini P., De Bellis L., Luvisi A. (2020). The *Xylella fastidiosa*-Resistant Olive Cultivar “Leccino” Has Stable Endophytic Microbiota during the Olive Quick Decline Syndrome (OQDS). Pathogens.

[122] Pacifico D., Squartini A., Crucitti D., Barizza E., Lo Schiavo F., Muresu R., Carimi F., Zottini M. (2019). The Role of the Endophytic Microbiome in the Grapevine Response to Environmental Triggers. Front. Plant Sci..

[123] Martini M., Musetti R., Grisan S., Polizzotto R., Borselli S., Pavan F., Osler R. (2009). DNA-Dependent Detection of the Grapevine Fungal Endophytes *Aureobasidium pullulans* and *Epicoccum nigrum*. Plant Dis..

[124] Bianco P.A., Marzachì C., Musetti R., Naor V. (2013). Perspectives of Endophytes as Biocontrol Agents in the Management of Phytoplasma Diseases. Phytopathog. Mollicutes.

[125] Bulgari D., Casati P., Crepaldi P., Daffonchio D., Quaglino F., Brusetti L., Bianco P.A. (2011). Restructuring of Endophytic Bacterial Communities in Grapevine Yellows-Diseased and Recovered *Vitis vinifera* L. Plants. Appl. Environ. Microbiol..

[126] Gamalero E., Marzachì C., Galetto L., Veratti F., Massa N., Bona E., Novello G., Glick B.R., Ali S., Cantamessa S. (2017). An 1-Aminocyclopropane-1-Carboxylate (ACC) Deaminase-Expressing Endophyte Increases Plant Resistance to Flavescence Dorée Phytoplasma Infection. Plant Biosyst..

[127] Ricciardi V., Crespan M., Maddalena G., Migliaro D., Brancadoro L., Maghradze D., Failla O., Toffolatti S.L., De Lorenzis G. (2024). Novel Loci Associated with Resistance to Downy and Powdery Mildew in Grapevine. Front. Plant Sci..

[128] Sprink T., Wilhelm R., Ricroch A., Eriksson D., Miladinović D., Sweet J., Van Laere K., Woźniak-Gientka E. (2023). Genome Editing in Biotech Regulations Worldwide. A Roadmap for Plant Genome Editing.

[129] Soriano J.M. (2020). Molecular Marker Technology for Crop Improvement. Agronomy.

[130] Ren C., Lin Y., Liang Z. (2022). CRISPR/Cas Genome Editing in Grapevine: Recent Advances, Challenges and Future Prospects. Fruit Res..

[131] Wang X., Tu M., Wang D., Liu J., Li Y., Li Z., Wang Y., Wang X. (2018). CRISPR/Cas9-Mediated Efficient Targeted Mutagenesis in Grape in the First Generation. Plant Biotechnol. J..

[132] Vezzulli S., Dolzani C., Nicolini D., Bettinelli P., Migliaro D., Gratl V., Stedile T., Zatelli A., Dallaserra M., Clementi S. (2019). Marker-Assisted Breeding for Downy Mildew, Powderey Mildew and Phylloxera Resistance at FEM. BIO Web Conf..

[133] Wan D.Y., Guo Y., Cheng Y., Hu Y., Xiao S., Wang Y., Wen Y.Q. (2020). CRISPR/Cas9-Mediated Mutagenesis of VvMLO3 Results in Enhanced Resistance to Powdery Mildew in Grapevine (*Vitis vinifera*). Hortic. Res..

[134] Akkurt M., Şenses I., Aktürk B., Tozlu I., Özer N., Uzun H.I. (2022). Marker Assisted Selection (MAS) for Downy Mildew Resistance in Grapevines Using Rpv3.1 Associated Markers. Not. Bot. Horti Agrobot. Cluj-Napoca.

[135] Fedorina J., Tikhonova N., Ukhatova Y., Ivanov R., Khlestkina E. (2022). Grapevine Gene Systems for Resistance to Gray Mold *Botrytis cinerea* and Powdery Mildew *Erysiphe necator*. Agronomy.

[136] Bettinelli P., Nicolini D., Costantini L., Stefanini M., Hausmann L., Vezzulli S. (2023). Towards Marker-Assisted Breeding for Black Rot Bunch Resistance: Identification of a Major QTL in the Grapevine Cultivar ‘Merzling’. Int. J. Mol. Sci..

[137] Giacomelli L., Zeilmaker T., Giovannini O., Salvagnin U., Masuero D., Franceschi P., Vrhovsek U., Scintilla S., Rouppe van der Voort J., Moser C. (2023). Simultaneous Editing of Two DMR6 Genes in Grapevine Results in Reduced Susceptibility to Downy Mildew. Front. Plant Sci..

[138] Rahman M.U., Liu X., Wang X., Fan B. (2024). Grapevine Gray Mold Disease: Infection, Defense and Management. Hortic. Res..

